# COLLABORATION FACTORS IN ADDRESSING ELDER IPV: COMMUNITY CENTERS AND DV SUPPORT AGENCIES IN JAPAN

**DOI:** 10.1093/geroni/igae098.2922

**Published:** 2024-12-31

**Authors:** Asako Katsumata, Kiyoko Fukushima, Noriko Tsukada

**Affiliations:** Japan Lutheran College, Tokyo, Japan; Japan Lutheran College, Tokyo, Japan; Nihon University, Tokyo, Japan

## Abstract

This study explores the determinants influencing collaboration between Community Comprehensive Support Centers (CCSCs) and Domestic Violence (DV) support agencies in addressing cases of older Intimate Partner Violence (IPV) in Japan. Utilizing data from a national survey conducted in November 2022, responses from 1,472 Community Social Workers (CSWs) out of 3,563 randomly selected CCSCs were analyzed. The study found that 5.8% of respondents frequently consulted with DV support organizations, 25.9% occasionally, 24.3% almost never, and 44.0% never at all. Logistic regression analysis, using gender, years of support experience, existence of a DV manual, number of husband-to-wife violence cases, and participation in training as independent variables, revealed that the presence of a domestic violence manual was the most significant factor influencing collaboration. CCSCs with a domestic violence manual were significantly more likely to consult DV support organizations (odds ratio = 2.158, 95% confidence interval: 1.425-3.266). These findings emphasize the importance of developing manuals on responding to domestic violence within CCSCs to facilitate collaboration with DV support agencies. This collaborative approach presents a viable option to enhance support mechanisms for older victims of intimate partner violence, indicating the potential impact of strategic interventions in addressing this critical societal issue.

